# Supplementary material for: Stable Carbenes as Structural Components of Partially Saturated Sulfur-Containing Heterocycles
Source: Molecules. 2022 Feb 22;27(5):1458. doi: 10.3390/molecules27051458 (PMC8911733; doi:10.3390/molecules27051458)
Supplement: Supplementary file 1 [file molecules-27-01458-s001.zip › molecules-ESI_1548459_2nd-proofreading.pdf]

Stable carbenes as structural components of partially saturated sulfur-containing heterocycles

A.B. Rozhenko,[1,2] Yu.S. Horbenko,[1,2] A.A. Kyrylchuk,[1]
E.V. Zarudnitskii,[1,2] S.S. Mykhaylychenko,[1] Yu.G. Shermolovich,[1]
A.V. Grafov[3,*]

[1] Institute of Organic Chemistry, National Academy of Sciences, Murmanska str. 5, Kyiv, 02094, Ukraine
[2] National Technical University of Ukraine "Igor Sikorsky Kyiv Polytechnic Institute", Prosp. Peremohy 37, Kyiv, 03056, Ukraine
[3] Department of chemistry, University of Helsinki, A.I.Virtasen aukio 1, 00560 Helsinki, Finland
** Correspondence: andriy.grafov@helsinki.fi*

Electronic Supplementary Information

Content:

	page
Table S1. Total energy values (E), zero-point energy correction (ZPE) and thermal correction to enthalpy (TCE) and Gibbs free energy (TCGFE), corrected energy values (E+ZPE, E+TCE and E+TCGFE, a.u.), the lowest vibration frequency for compounds of **1-8** (RI-SCS-MP2/cc-pVTZ).	S2
Figure S1. Model linear structures **9** and **10**.	S5
Figure S2. Dependence of C-S bond lengths in model compounds **9a-g**, **10a-o** (a), **9a-g** (b) and **10a-o** (c). Linear approximations and coefficients of determination are indicated for each plot.	S6
Table S2. Bond lengths and total NBO charges on carbene moieties for compounds **9a-g**, **10a-o**.	S7
Cartesian coordinates for the equilibrium structures **1-6** (SCS-MP2/cc-pVTZ) for compounds of **1-8** (RI-SCS-MP2/cc-pVTZ).	S8

Table S1. Total energy values (E), zero-point energy correction (ZPE) and thermal correction to enthalpy (TCE) and Gibbs free energy (TCGFE), corrected energy values (E+ZPE, E+TCE and E+TCGFE, a.u.), the lowest vibration frequency for compounds of **1-8** (RI-SCS-MP2/cc-pVTZ).

Structure	Energy, a.u.	ZPE, a.u.	E+ZPE, a.u.	TCE a.u.	E+ TCE, a.u.	TCGFE a.u.	E+ TCGFE, a.u.	ν, cm^{-1}
**1a**	-990.313249	0.119186	-990.194063	0.122179	-990.191070	0.081135	-990.232114	118.3
**1b**	-1084.769533	0.164850	-1084.604683	0.174061	-1084.595472	0.121474	-1084.648059	94.0
**1c**	-1179.228448	0.209825	-1179.018623	0.213742	-1179.014706	0.160149	-1179.068299	124.6
**1d**	-1382.140199	0.141429	-1381.998770	0.147949	-1381.992250	0.095826	-1382.044373	66.6
**1e**	-911.857324	0.062894	-911.794431	0.065805	-911.791520	0.030767	-911.826557	158.6
**1f**	-1830.060018	0.043081	-1830.016936	0.049366	-1830.010652	0.007770	-1830.052248	92.6
**1g**	-1110.095875	0.046403	-1110.049472	0.051735	-1110.044140	0.011872	-1110.084003	34.7
**1h**	-1289.740930	0.244792	-1289.496138	0.249838	-1289.491092	0.190585	-1289.550345	44.3
**1i**	-1587.582369	0.215216	-1587.367152	0.224437	-1587.357931	0.156580	-1587.425789	25.7
**1j**	-1377.440115	0.194344	-1377.245770	0.200903	-1377.239212	0.142879	-1377.297235	32.8
**1k**	-1234.483540	0.227065	-1234.256475	0.231602	-1234.251939	0.175296	-1234.308244	52.4
**1l**	-1383.413668	0.212780	-1383.200888	0.219223	-1383.194444	0.158636	-1383.255031	22.8
**1m**	-1278.337537	0.202117	-1278.135420	0.207267	-1278.130270	0.151838	-1278.185699	41.4
**2a**	-742.307593	0.180813	-742.126780	0.182895	-742.124698	0.137368	-742.170224	91.3
**2b**	-836.767447	0.226033	-836.541415	0.228589	-836.538858	0.176942	-836.590506	80.9
**2c**	-875.980001	0.253755	-875.726245	0.256664	-875.723337	0.201470	-875.778530	82.8

Table S1. Continued.

Struc-ture	Energy, a.u.	ZPE, a.u.	E+ZPE, a.u.	TCE a.u.	E+ TCE, a.u.	TCGFE a.u.	E+ TCGFE, a.u.	ν, cm^{-1}
**3a**	-856.624248	0.213283	-856.410964	0.216412	-856.407835	0.164574	-856.459673	68.5
**3b**	-895.856691	0.241323	-895.615368	0.244765	-895.611926	0.189092	-895.667600	43.9
**4a**	-663.864978	0.123226	-663.741752	0.125462	-663.739516	0.085896	-663.779082	145.8
**4b**	-758.326977	0.169261	-758.157716	0.171647	-758.155330	0.126624	-758.200353	99.3
**4c**	-852.784680	0.214228	-852.570452	0.217166	-852.567514	0.165934	-852.618746	87.4
**4d**	-1055.690500	0.145203	-1055.545296	0.151202	-1055.539298	0.098941	-1055.591559	29.0
**4e**	-703.081629	0.151224	-702.930405	0.153533	-702.928096	0.110790	-702.970838	108.3
**4f**	-797.543278	0.197128	-797.346150	0.199695	-797.343583	0.151426	-797.391852	95.5
**4g**	-891.999824	0.242159	-891.757665	0.245325	-891.754499	0.190893	-891.808931	97.3
**4h**	-1094.904191	0.173344	-1094.730847	0.179356	-1094.724835	0.124894	-1094.779297	44.5
**5a**	-719.091305	0.138546	-718.952759	0.141600	-718.949705	0.098412	-718.992893	97.5
**5b**	-813.554630	0.184587	-813.370043	0.187858	-813.366773	0.139124	-813.415506	88.4
**5c**	-908.010980	0.229795	-907.781185	0.233563	-907.777416	0.178899	-907.832080	89.3
**6a**	-834.357303	0.180147	-834.177156	0.183546	-834.173757	0.134633	-834.222669	86.8
**6a’**	-873.574131	0.208151	-873.365980	0.211735	-873.362396	0.159463	-873.414668	81.9
**6b**	-850.377187	0.168747	-850.208440	0.172531	-850.204656	0.123820	-850.253366	96.0
**6b’**	-889.595555	0.196531	-889.399024	0.200488	-889.395068	0.148574	-889.446981	90.9
**6c**	-905.602175	0.183915	-905.418260	0.188383	-905.413792	0.136492	-905.465683	82.0
**6d**	-854.208366	0.167219	-854.041147	0.171335	-854.037031	0.121032	-854.087334	26.6
**6e**	-1176.817020	0.163985	-1176.653035	0.168974	-1176.648046	0.117197	-1176.699823	46.1

Table S1. Continued.

Structure	Energy, a.u.	ZPE, a.u.	E+ZPE, a.u.	TCE a.u.	E+ TCE, a.u.	TCGFE a.u.	E+ TCGFE, a.u.	ν, cm^{-1}
**7a**	-306.520371	0.172342	-306.348028	0.174047	-306.346324	0.130981	-306.389390	75.9
**7b**	-509.423546	0.103449	-509.320097	0.107960	-509.315586	0.063314	-509.360232	14.3
**7c**	-237.372399	0.007088	-237.365311	0.010687	-237.361711	-0.016698	-237.389096	681.4
**7d**	-304.147740	0.127768	-304.019972	0.129587	-304.018153	0.090745	-304.056995	104.9
**7e**	-305.342896	0.151245	-305.191651	0.152474	-305.190422	0.111899	-305.230997	116.0
**8a**	-835.575879	0.204950	-835.370929	0.207039	-835.368841	0.158259	-835.417620	68.3
**8b**	-855.429068	0.192093	-855.236975	0.194812	-855.234256	0.145565	-855.283504	54.0
**8c**	-1178.035416	0.188695	-1177.846721	0.192184	-1177.843231	0.141543	-1177.893873	47.3
**8d**	-851.593795	0.193053	-851.400742	0.195628	-851.398167	0.146947	-851.446848	69.8
**8e**	-890.811149	0.221129	-890.590021	0.223809	-890.587341	0.172146	-890.639003	81.7

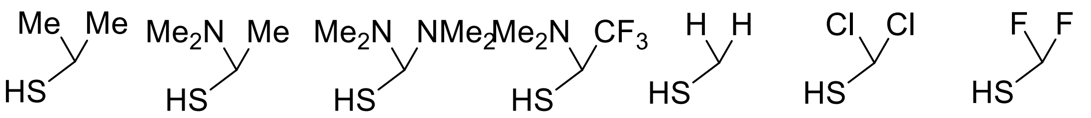

9 a **9b** **9c** **9d** **9e** **9f** **9g**

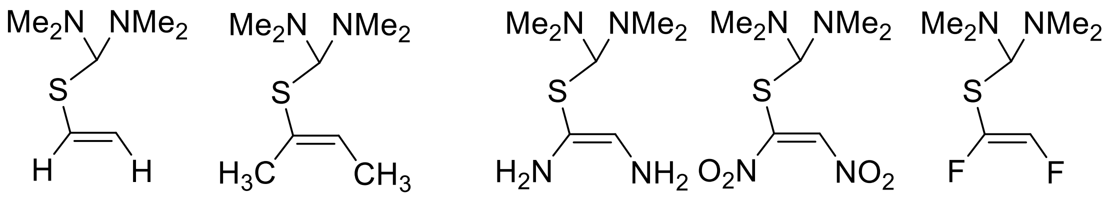

10a **10b** **10c** **10d** **10e**

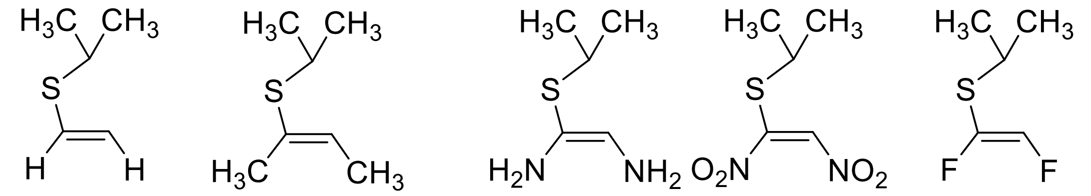

10f **10g** **10h** **10i** **10j**

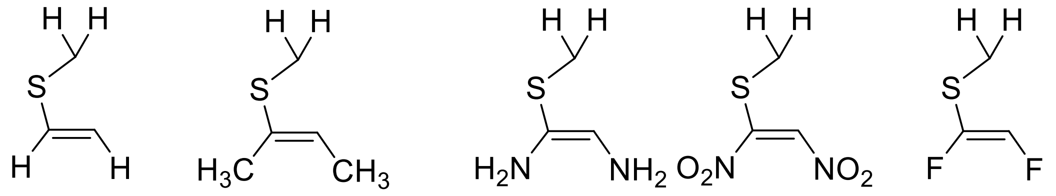

10k **10l** **10m** **10n** **10o**

Figure S1. Model linear structures **9** and **10**.

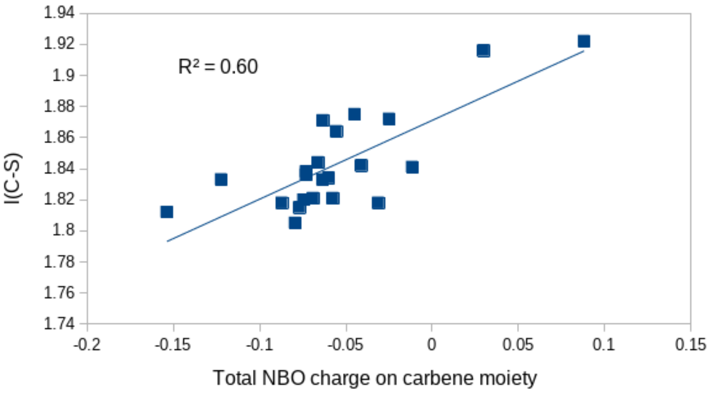

a

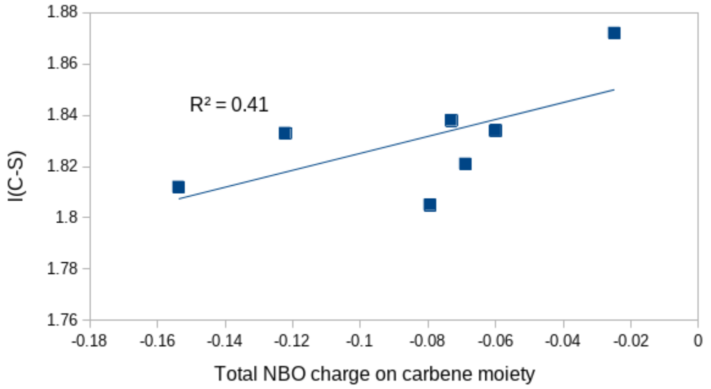

b

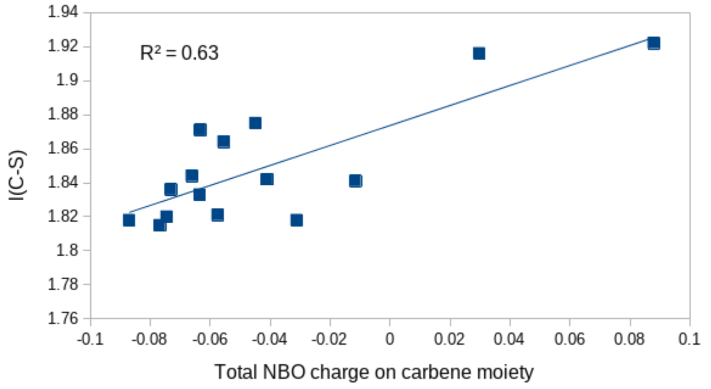

c

Figure S2. Dependence of C-S bond lengths in model compounds **9a-g**, **10a-o** (a), **9a-g** (b) and **10a-o** (c). Linear approximations and coefficients of determination are indicated for each plot.

Table S2. Bond lengths and total NBO charges on carbene moieties for compounds **9a-g**, **10a-o**.

Compd.	l(C-S)	Carbene charge
**9a**	1.834	-0.06
**9b**	1.838	-0.0731
**9c**	1.872	-0.0249
**9d**	1.833	-0.1222
**9e**	1.821	-0.0689
**9f**	1.812	-0.1537
**9g**	1.805	-0.0794
**10a**	1.864	-0.0555
**10b**	1.871	-0.0633
**10c**	1.875	-0.0449
**10d**	1.922	0.088
**10e**	1.916	0.0298
**10f**	1.833	-0.0635
**10g**	1.836	-0.0731
**10h**	1.844	-0.0661
**10i**	1.841	-0.0115
**10j**	1.842	-0.041
**10k**	1.815	-0.0768
**10l**	1.818	-0.087
**10m**	1.82	-0.0745
**10n**	1.818	-0.0311
**10o**	1.821	-0.0575

Cartesian coordinates for the equilibrium structures 1-6 (SCS-MP2/cc-pVTZ) for compounds of 1-8 (RI-SCS-MP2/cc-pVTZ).

1a

S	-5.99520	0.10950	0.27843
C	-6.42564	-0.79476	-1.16612
C	-5.55377	-0.77067	-2.18678
S	-4.08112	0.16238	-1.96229
C	-4.19693	0.14417	-0.12315
C	-3.52280	-1.11199	0.42308
C	-3.56540	1.41128	0.44623
H	-7.37695	-1.30496	-1.19436
H	-5.71868	-1.25915	-3.13562
H	-2.46428	-1.10859	0.15707
H	-3.98520	-2.00590	0.00699
H	-3.61983	-1.14050	1.50981
H	-4.05032	2.30188	0.05301
H	-2.50471	1.44161	0.19265
H	-3.65046	1.40996	1.53390

1b

S	-5.96252	-0.00793	0.32788
C	-6.52577	-0.70156	-1.17950
C	-5.68107	-0.71275	-2.22215
S	-4.10108	0.01128	-2.00258
C	-4.16622	0.05144	-0.11756
N	-3.46845	-1.03439	0.52975
C	-3.61134	1.40792	0.32745
H	-7.52883	-1.10182	-1.20886
H	-5.92516	-1.12694	-3.18960
H	-4.26660	2.19967	-0.02189
H	-2.61804	1.58018	-0.08381
H	-3.55188	1.43557	1.41624
C	-2.01033	-0.95308	0.41647
C	-3.91874	-2.36709	0.13385
H	-1.62927	-0.06694	0.91514
H	-1.66824	-0.95609	-0.62729
H	-1.58688	-1.82326	0.91415
H	-4.99200	-2.45218	0.27527
H	-3.42729	-3.09734	0.77470
H	-3.67542	-2.59445	-0.91147

1c

S	-1.35661	0.85915	-0.61958
C	-0.51988	0.99639	-2.14732
C	0.73226	0.52411	-2.24845
S	1.49483	-0.17598	-0.84131
C	-0.03712	-0.16603	0.26938
N	-0.55529	-1.49687	0.48994
C	0.37061	-2.35157	1.24393
C	-0.97236	-2.19756	-0.72342
N	0.32326	0.41275	1.54369
C	-0.79758	0.41697	2.49216
C	0.89684	1.75682	1.48013
H	-1.05005	1.45798	-2.96784
H	1.31202	0.56533	-3.15928
H	1.27940	-2.57329	0.66988
H	0.65513	-1.87661	2.17623
H	-0.13847	-3.29035	1.45698
H	-0.12353	-2.42272	-1.38096
H	-1.43990	-3.13438	-0.42430
H	-1.70049	-1.60649	-1.26956
H	-1.22588	-0.57499	2.58107
H	-1.58751	1.11472	2.18283
H	-0.41963	0.73782	3.46168
H	1.77079	1.76885	0.83777
H	0.17559	2.49580	1.11044
H	1.19897	2.03457	2.48886

1d

S	-1.52530	-0.93271	0.38033
C	-2.09244	-0.89097	-1.27577
C	-1.18712	-0.88796	-2.26344
S	0.51220	-0.86037	-1.84672
C	0.27250	-0.56167	-0.03450
C	1.10532	-1.63862	0.71112
N	0.72418	0.76626	0.28555
C	0.84601	1.07962	1.71519
C	-0.06341	1.80964	-0.37442
H	-3.16023	-0.88496	-1.43835
H	-1.43831	-0.88074	-3.31399
H	1.62500	0.48717	2.18104
H	-0.09529	0.93614	2.25926
H	1.12853	2.12713	1.79094
H	-0.12994	1.61061	-1.43951
H	-1.07524	1.88577	0.04070
H	0.44923	2.75868	-0.23141
F	2.41465	-1.38657	0.59609
F	0.87993	-2.86040	0.23538
F	0.80973	-1.67603	2.02251

1e

S	-1.58354	-0.23084	0.06530
C	-0.50431	-0.79982	-1.20791
C	0.75329	-0.33624	-1.20420
S	1.19841	0.79463	0.07350
C	-0.20110	0.29655	1.13056
H	-0.90558	-1.45562	-1.96578
H	1.48813	-0.57325	-1.95872
H	0.10008	-0.52564	1.77704
H	-0.51717	1.14923	1.72577

1f

S	0.43420	-0.28891	-1.47630
C	-0.02530	1.20082	-0.67106
C	-0.02530	1.20082	0.67106
S	0.43420	-0.28891	1.47630
C	0.11798	-1.30954	0.00000
Cl	1.19594	-2.72440	0.00000
Cl	-1.58913	-1.90376	0.00000
H	-0.27130	2.05694	-1.28084
H	-0.27130	2.05694	1.28084

1g

S	0.43420	-0.28891	-1.47630
C	-0.02530	1.20082	-0.67106
C	-0.02530	1.20082	0.67106
S	0.43420	-0.28891	1.47630
C	0.11798	-1.30954	0.00000
Cl	1.19594	-2.72440	0.00000
Cl	-1.58913	-1.90376	0.00000
H	-0.27130	2.05694	-1.28084
H	-0.27130	2.05694	1.28084

1h

S	-5.88023	0.04670	0.43246
C	-6.46195	-0.61263	-1.08846
C	-5.61949	-0.64043	-2.13970
S	-3.99518	0.03756	-1.92413
C	-4.05051	0.08871	-0.04409
N	-3.37243	-1.03224	0.56977
C	-1.92748	-1.03360	0.30943
C	-3.91933	-2.34292	0.23011
N	-3.42366	1.31029	0.40702
C	-3.41117	1.42310	1.87078
C	-3.95570	2.54939	-0.16096
N	-7.80915	-1.00276	-1.12968
N	-6.02783	-1.17050	-3.39759
H	-1.70487	-1.20624	-0.75107
H	-1.48660	-0.08904	0.60852
H	-1.48400	-1.83972	0.89219
H	-3.78050	-2.58625	-0.83098
H	-3.39621	-3.08809	0.82780
H	-4.97631	-2.38614	0.47329
H	-3.00151	0.52443	2.31785
H	-4.41947	1.58869	2.27447
H	-2.79283	2.27816	2.13983
H	-3.89932	2.52998	-1.24353
H	-4.99672	2.72680	0.13421
H	-3.34117	3.36901	0.20897
H	-7.98822	-1.47593	-2.00806
H	-8.05372	-1.60317	-0.35315
H	-5.33942	-1.82371	-3.75322
H	-6.12414	-0.43547	-4.08914

1i

S	-5.96985	-0.62557	0.53428
C	-6.50461	-0.65139	-1.10882
C	-5.59837	-0.62912	-2.10816
S	-3.90276	-0.52993	-1.71422
C	-4.17915	-0.08364	0.10915
N	-3.29700	-0.81294	0.97512
C	-1.88931	-0.43248	0.78377
C	-3.41001	-2.27274	0.92971
N	-3.99394	1.31959	0.28609
C	-4.11870	1.75669	1.68444
C	-4.80523	2.18362	-0.57289
N	-7.96262	-0.68154	-1.29443
N	-5.94171	-0.68532	-3.51839
O	-8.48785	-1.78702	-1.31108
O	-8.52072	0.40585	-1.36156
O	-4.99526	-0.72640	-4.30327
O	-7.13144	-0.69940	-3.82010
H	-1.50982	-0.76550	-0.19140
H	-1.76970	0.64234	0.85527
H	-1.30145	-0.91616	1.56139
H	-3.11776	-2.67998	-0.04477
H	-2.74702	-2.67524	1.69309
H	-4.42376	-2.59028	1.14908
H	-3.49049	1.15344	2.32977
H	-5.15496	1.69163	2.03696
H	-3.80089	2.79608	1.73824
H	-4.62196	1.96101	-1.61921
H	-5.87686	2.09119	-0.36001
H	-4.50346	3.21202	-0.38568

1j

N	-0.00606	0.32153	-1.27304
C	1.37486	0.10874	-1.31145
C	1.90263	-0.54010	-0.26333
S	0.68916	-1.02979	0.91510
C	-0.70562	-0.42195	-0.21982
N	-1.46808	-1.51226	-0.79748
C	-2.21980	-2.28122	0.19864
C	-0.66698	-2.43393	-1.60345
N	-1.61475	0.41314	0.55866
C	-2.72889	0.90589	-0.26254
C	-0.95535	1.56824	1.17157
H	-0.48572	0.27289	-2.16084
H	1.93284	0.51827	-2.14043
H	2.94911	-0.72672	-0.09317
H	-1.55023	-2.81940	0.88224
H	-2.85711	-1.62633	0.78193
H	-2.83358	-3.01150	-0.32708
H	0.05145	-2.99663	-0.99551
H	-1.34575	-3.13653	-2.08452
H	-0.12179	-1.90370	-2.37904
H	-3.26614	0.08069	-0.71780
H	-2.38761	1.58573	-1.05496
H	-3.40720	1.45846	0.38595
H	-0.16451	1.24398	1.83978
H	-0.53069	2.24641	0.42143
H	-1.70264	2.10856	1.75098

1k

S	0.50918	-0.25519	1.14712
C	-0.34296	-1.72929	1.59561
C	-1.43601	-2.06689	0.88941
S	-1.94830	-1.02720	-0.42781
C	-0.85030	0.43611	0.03111
N	-0.22317	1.01932	-1.13486
C	-1.20579	1.60679	-2.05445
C	0.66518	0.13456	-1.88967
N	-1.64019	1.45218	0.68600
C	-0.86456	2.64962	1.03081
C	-2.37631	0.99806	1.86195
N	0.27661	-2.48424	2.60849
H	-2.02687	-2.94763	1.09898
H	-1.81919	0.83596	-2.54061
H	-1.86101	2.28932	-1.52514
H	-0.66393	2.14884	-2.82795
H	0.12853	-0.71405	-2.32967
H	1.11008	0.72393	-2.69019
H	1.45793	-0.24695	-1.25557
H	-0.34800	3.03101	0.15689
H	-0.12533	2.44527	1.81580
H	-1.55869	3.40396	1.39851
H	-3.01530	0.15925	1.60571
H	-1.70650	0.70760	2.68193
H	-3.00215	1.82145	2.20309
H	-0.28542	-3.28306	2.86973
H	0.47215	-1.92713	3.42983

1l

S	-1.47203	-0.19795	-0.69146
C	-2.02514	0.82459	0.58598
C	-1.11742	1.57099	1.23821
S	0.57710	1.49482	0.83146
C	0.39160	0.02666	-0.35725
N	1.04199	0.27548	-1.61675
C	2.49972	0.40100	-1.47923
C	0.53382	1.41957	-2.37534
N	0.96590	-1.13862	0.25761
C	0.94855	-2.32104	-0.61457
C	0.40894	-1.49098	1.56305
N	-1.50620	2.48711	2.29974
O	-0.60402	3.16620	2.78990
O	-2.68853	2.52753	2.62576
H	-3.07747	0.85830	0.82555
H	2.77744	1.31655	-0.94002
H	2.90905	-0.45193	-0.95016
H	2.93019	0.44990	-2.47784
H	0.71633	2.36999	-1.86022
H	1.04978	1.43409	-3.33377
H	-0.53107	1.31842	-2.55596
H	1.40365	-2.09554	-1.57234
H	-0.07197	-2.68546	-0.78592
H	1.51573	-3.10824	-0.12098
H	0.49142	-0.65457	2.24973
H	-0.64050	-1.80278	1.49298
H	0.99080	-2.32051	1.96048

1m

S	0.93491	0.75944	-1.11818
C	2.20522	0.33660	0.00964
C	1.86615	-0.39332	1.07645
S	0.20579	-0.86483	1.36448
F	2.74894	-0.79680	2.00282
H	3.21560	0.66274	-0.18325
C	-0.51330	0.22319	-0.01615
N	-1.44922	-0.50874	-0.82825
C	-2.64341	-0.91932	-0.07882
C	-0.90807	-1.67057	-1.53474
N	-1.18675	1.34580	0.58934
C	-1.84814	2.21189	-0.39652
C	-0.34847	2.16804	1.46086
H	-2.40561	-1.68907	0.66817
H	-3.08903	-0.07072	0.42714
H	-3.35833	-1.33938	-0.78428
H	-0.59586	-2.46274	-0.84386
H	-1.69403	-2.05791	-2.18107
H	-0.05947	-1.38554	-2.14644
H	-2.51530	1.63378	-1.02610
H	-1.12223	2.73048	-1.03470
H	-2.42314	2.95828	0.14909
H	0.08720	1.56337	2.24971
H	0.45121	2.67307	0.90539
H	-0.98507	2.92347	1.91818

2a

N	0.70906	-0.32661	1.23119
C	1.66108	-1.35291	1.20844
C	2.04071	-1.77938	-0.00427
S	1.19616	-0.93141	-1.30117
C	0.01459	-0.13780	-0.04748
N	-1.30509	-0.73971	0.01495
C	-1.29223	-2.18353	0.25642
C	-2.14304	-0.45243	-1.14935
C	-0.07836	1.35129	-0.37490
H	0.05119	-0.36402	1.99880
H	2.05888	-1.70187	2.14977
H	2.79611	-2.51637	-0.21528
H	-0.70433	-2.41842	1.13888
H	-0.88100	-2.74197	-0.59277
H	-2.31672	-2.50925	0.43027
H	-1.69102	-0.80743	-2.08572
H	-2.34367	0.61150	-1.23121
H	-3.09513	-0.96340	-1.01807
H	-0.35476	1.52536	-1.41329
H	0.89470	1.79849	-0.19742
H	-0.81753	1.82154	0.27641

2b

N	-0.87290	-0.85880	0.82386
C	-2.22991	-0.98637	0.51409
C	-2.59650	-0.58779	-0.71263
S	-1.25423	0.06930	-1.64406
C	-0.10131	-0.07015	-0.14264
N	1.13347	-0.72813	-0.55773
N	0.23695	1.22268	0.42124
C	0.91521	-2.06172	-1.12221
C	2.07789	-0.85453	0.56056
C	-0.92148	1.99315	0.87427
C	1.03761	2.05405	-0.48233
H	-0.65904	-0.61103	1.77963
H	-2.88157	-1.43933	1.24649
H	-3.57909	-0.67881	-1.14289
H	1.88351	-2.45575	-1.42754
H	0.27206	-2.00469	-1.99409
H	0.46684	-2.74898	-0.39447
H	2.29173	0.11596	0.99562
H	3.00153	-1.28379	0.17506
H	1.69749	-1.51905	1.34814
H	-1.52161	1.42176	1.57661
H	-1.56255	2.30364	0.04061
H	-0.55474	2.88227	1.38493
H	1.92758	1.52215	-0.79902
H	1.32583	2.95817	0.05238
H	0.46725	2.34579	-1.37387

**2c**				**3a**				**3b**			
N	-0.61285	-0.59159	0.53197	O	-5.20261	-0.57712	-0.64651	O	0.02403	-0.56294	0.31212
C	-1.81120	-1.20273	0.21107	C	-5.57944	-0.69739	-1.95358	C	-1.33787	-0.67350	0.43921
C	-2.29174	-0.96973	-1.02262	C	-4.72335	-0.30186	-2.90034	C	-2.08100	-0.26514	-0.59648
S	-1.20278	0.04419	-1.96266	S	-3.21122	0.28960	-2.23443	S	-1.12550	0.31657	-1.94959
C	0.11938	-0.00762	-0.59062	C	-3.85706	-0.07715	-0.47642	C	0.46224	-0.06302	-0.96786
N	1.24987	-0.77832	-1.09211	N	-3.00900	-1.05152	0.15413	N	1.24549	-1.04123	-1.67463
N	0.58654	1.31458	-0.23261	N	-3.93990	1.10099	0.33608	N	1.25133	1.10987	-0.72078
C	0.92777	-2.16505	-1.40292	H	-6.56573	-1.11158	-2.08974	C	-1.77363	-1.24043	1.74551
C	2.50567	-0.71196	-0.33816	C	-2.93958	-2.32040	-0.57225	C	0.53719	-2.30325	-1.89230
C	-0.43481	2.15381	0.39229	C	-3.39559	-1.31871	1.54823	C	2.52863	-1.32122	-1.01212
C	1.17884	2.03152	-1.36463	H	-2.18993	-2.94547	-0.09004	C	0.59261	2.09044	0.13891
C	0.09502	-0.93434	1.75067	H	-2.63770	-2.14892	-1.60075	C	1.71674	1.74453	-1.95378
H	-2.29586	-1.79759	0.97196	H	-3.89729	-2.85439	-0.56182	H	-3.15673	-0.29159	-0.63337
H	-3.21988	-1.33192	-1.42822	H	-3.37249	-0.40599	2.13275	H	-2.85905	-1.30094	1.78427
H	1.77557	-2.60469	-1.92832	H	-2.67777	-2.02631	1.96052	H	-1.42421	-0.61616	2.56892
H	0.05654	-2.21361	-2.04961	H	-4.39802	-1.75550	1.61913	H	-1.35790	-2.23910	1.88526
H	0.73468	-2.76711	-0.50452	C	-4.91465	2.07942	-0.14088	H	1.15696	-2.93423	-2.52721
H	2.68449	0.29730	0.01598	C	-2.63819	1.73593	0.54141	H	-0.40627	-2.12227	-2.39775
H	3.31652	-1.00352	-1.00752	H	-5.90007	1.62879	-0.19575	H	0.34626	-2.83638	-0.95273
H	2.52507	-1.39116	0.52224	H	-4.64594	2.48651	-1.12374	H	3.11203	-0.41309	-0.91073
H	-0.81565	1.68581	1.29477	H	-4.94558	2.89618	0.57831	H	3.07425	-2.03007	-1.63334
H	-1.27701	2.34948	-0.28253	H	-1.91868	1.00644	0.89935	H	2.38957	-1.76149	-0.01823
H	0.03197	3.10164	0.65775	H	-2.75344	2.52353	1.28449	H	0.33931	1.63972	1.09307
H	1.91309	1.40908	-1.86507	H	-2.25378	2.18656	-0.38349	H	-0.31483	2.50165	-0.32101
H	1.66385	2.93288	-0.99166	H	-4.90419	-0.34275	-3.96064	H	1.29442	2.90404	0.31498
H	0.41508	2.32760	-2.09600					H	2.20881	1.01344	-2.58738
H	0.84754	-0.17359	1.94716					H	2.42510	2.52865	-1.69057
H	0.58477	-1.91206	1.70748					H	0.89000	2.19932	-2.51590
H	-0.61145	-0.93877	2.58120								

4a

N	-1.16101	0.02840	-0.39182
N	-1.77670	-1.03463	0.28069
C	-0.96572	-1.54352	1.13684
S	0.59753	-0.74794	1.32253
C	-0.13210	0.69603	0.43448
C	0.89633	1.36942	-0.46281
C	-0.74216	1.67496	1.43794
H	-1.88735	0.67814	-0.67250
H	-1.22037	-2.43231	1.69184
H	1.69436	1.81613	0.13021
H	0.41366	2.16684	-1.03128
H	1.32035	0.64960	-1.15817
H	0.02604	2.08318	2.09443
H	-1.49351	1.17520	2.04830
H	-1.21548	2.50169	0.90171

4b

N	-1.44718	-0.09238	-0.12969
N	-1.91742	-1.34722	0.24446
C	-1.05286	-1.96174	0.97096
S	0.48828	-1.16410	1.23871
C	0.01713	0.07133	-0.10926
N	0.58485	-0.18694	-1.41199
C	0.30676	-1.52145	-1.94453
C	2.01622	0.09728	-1.51070
C	0.36395	1.47615	0.37955
H	-1.82702	0.15048	-1.03672
H	-1.27401	-2.92548	1.40176
H	-0.75825	-1.73232	-1.90922
H	0.83673	-2.30780	-1.39345
H	0.62827	-1.54693	-2.98437
H	2.60974	-0.50468	-0.80887
H	2.21665	1.14979	-1.33482
H	2.34243	-0.14154	-2.52114
H	1.41284	1.56798	0.65403
H	-0.24486	1.69452	1.25111
H	0.14035	2.19651	-0.40890

4c

N	0.65340	-0.53398	0.98208
N	1.01237	-1.81732	0.60765
C	0.23725	-2.26294	-0.31875
S	-0.94921	-1.16299	-0.98859
C	-0.14507	0.23611	0.01023
N	-1.10809	1.03894	0.71537
C	-1.86831	0.32616	1.74083
C	-2.02525	1.74287	-0.18168
N	0.61835	1.10012	-0.88239
C	1.66331	0.40200	-1.62930
C	1.21256	2.25209	-0.19361
H	1.46912	-0.03170	1.30569
H	0.33220	-3.27629	-0.67617
H	-1.19529	-0.16100	2.43699
H	-2.53606	-0.42908	1.30760
H	-2.47058	1.05913	2.27575
H	-2.71229	1.04576	-0.68215
H	-1.47179	2.28749	-0.93918
H	-2.61852	2.44160	0.40632
H	2.12800	1.11655	-2.30665
H	1.23413	-0.40135	-2.21973
H	2.44540	-0.01313	-0.97888
H	2.00316	1.96395	0.51522
H	0.45483	2.80850	0.34601
H	1.66733	2.89519	-0.94564

4d

N	-1.43863	0.21272	0.34859
N	-2.40266	-0.74933	0.64796
C	-2.07607	-1.43894	1.67520
S	-0.62384	-0.98907	2.55695
C	-0.36259	0.37923	1.33548
N	0.92216	0.22186	0.69171
C	-0.56811	1.72869	2.07269
F	0.29995	1.90341	3.07435
F	-1.80169	1.83210	2.57998
F	-0.40963	2.75268	1.21118
C	2.09904	0.27957	1.55124
C	1.13003	0.96440	-0.55034
H	-1.90871	1.07383	0.10282
H	-2.67470	-2.27513	2.00076
H	1.93357	-0.30200	2.45629
H	2.38465	1.29778	1.83734
H	2.93135	-0.16937	1.01013
H	1.35550	2.02459	-0.39143
H	0.25456	0.86914	-1.18496
H	1.97475	0.50961	-1.06679

4e

N	0.56381	0.78499	0.50164
N	1.35029	-0.14013	1.18990
C	1.07820	-1.33819	0.81232
S	-0.22229	-1.51704	-0.36074
C	-0.73048	0.20521	0.07276
C	-1.77997	0.15772	1.18736
C	-1.25636	0.92593	-1.16212
C	0.49281	2.04372	1.23715
H	1.64893	-2.18180	1.16635
H	-2.64505	-0.41238	0.85021
H	-2.11184	1.16414	1.44758
H	-1.37467	-0.32249	2.07775
H	-2.14207	0.42147	-1.54862
H	-0.49216	0.95473	-1.93499
H	-1.54335	1.94649	-0.90486
H	1.50844	2.40272	1.37842
H	0.01604	1.94499	2.21653
H	-0.05443	2.77235	0.64206

4f

N	-0.98991	-0.11429	-0.07967
N	-1.29453	-1.38385	0.37258
C	-0.26574	-1.94833	0.90384
S	1.23807	-1.04364	0.87051
C	0.43219	0.13634	-0.36278
N	0.75845	-0.11713	-1.74683
C	0.47303	-1.47838	-2.19826
C	2.12024	0.25633	-2.12436
C	0.79774	1.56685	0.03574
C	-1.96699	0.41720	-1.02054
H	-0.33816	-2.92132	1.36245
H	-0.54325	-1.75974	-1.94006
H	1.15742	-2.21468	-1.75947
H	0.57681	-1.50772	-3.28221
H	2.87871	-0.28310	-1.53985
H	2.27796	1.32478	-2.01070
H	2.26269	0.00673	-3.17429
H	1.87395	1.69780	0.12771
H	0.33900	1.78242	0.99590
H	0.42626	2.26597	-0.71445
H	-1.76886	1.47628	-1.16971
H	-2.94974	0.30178	-0.57103
H	-1.95402	-0.07894	-1.99362

4g

N	1.05465	0.31695	-0.10776
N	1.87489	-0.19658	0.86243
C	1.20012	-0.80182	1.78176
S	-0.52812	-0.92527	1.54641
C	-0.29169	-0.27275	-0.22452
N	-1.23542	0.76231	-0.56293
C	-1.09152	1.99710	0.20718
C	-2.62500	0.30409	-0.49678
N	-0.45692	-1.38073	-1.14682
C	0.48306	-2.48016	-0.96315
C	-0.60422	-1.06949	-2.57284
C	1.74614	0.70199	-1.32714
H	1.69132	-1.21438	2.64834
H	-0.09384	2.40678	0.09162
H	-1.28252	1.84149	1.27597
H	-1.81762	2.71131	-0.17912
H	-3.26624	1.06364	-0.94199
H	-2.94479	0.14881	0.54286
H	-2.74400	-0.62869	-1.03754
H	0.18003	-3.29690	-1.61775
H	0.45530	-2.83667	0.06229
H	1.51632	-2.20745	-1.21212
H	0.35794	-0.94820	-3.08230
H	-1.18012	-0.16047	-2.70520
H	-1.13100	-1.89837	-3.04819
H	1.04277	1.23251	-1.96583
H	2.16188	-0.14714	-1.87540
H	2.55845	1.37221	-1.05513

4h

N	-1.43863	0.21272	0.34859
N	-2.40266	-0.74933	0.64796
C	-2.07607	-1.43894	1.67520
S	-0.62384	-0.98907	2.55695
C	-0.36259	0.37923	1.33548
N	0.92216	0.22186	0.69171
C	-0.56811	1.72869	2.07269
F	0.29995	1.90341	3.07435
F	-1.80169	1.83210	2.57998
F	-0.40963	2.75268	1.21118
C	2.09904	0.27957	1.55124
C	1.13003	0.96440	-0.55034
H	-1.90871	1.07383	0.10282
H	-2.67470	-2.27513	2.00076
H	1.93357	-0.30200	2.45629
H	2.38465	1.29778	1.83734
H	2.93135	-0.16937	1.01013
H	1.35550	2.02459	-0.39143
H	0.25456	0.86914	-1.18496
H	1.97475	0.50961	-1.06679

5a

S	-2.25064	2.22954	-1.31828
C	-2.83209	3.60815	-0.26686
N	-1.52272	4.09498	0.21039
N	-0.50785	3.91604	-0.69000
N	-0.67899	2.99529	-1.52639
C	-1.45154	5.37757	0.90012
H	-2.11285	5.35036	1.76335
H	-1.72649	6.21865	0.25995
H	-0.43098	5.51300	1.24518
C	-3.58327	4.65087	-1.09917
H	-4.46330	4.19082	-1.54786
H	-2.94829	5.03079	-1.89925
H	-3.91236	5.48539	-0.47741
C	-3.67524	3.09828	0.89720
H	-3.11299	2.37702	1.48532
H	-4.58517	2.62682	0.52663
H	-3.97394	3.93031	1.53707

5b

S	-2.08261	2.47782	-1.69529
C	-2.78026	3.73410	-0.51949
N	-1.52036	4.37497	-0.13053
N	-0.40151	3.61136	-0.22769
N	-0.48561	2.60826	-0.98635
C	-1.50931	5.36575	0.93681
H	-1.81543	4.94940	1.89707
H	-2.18599	6.17571	0.67336
H	-0.49856	5.75628	1.00810
C	-3.66657	4.72192	-1.27986
H	-4.44636	4.21057	-1.83999
H	-3.04803	5.27584	-1.98027
H	-4.14008	5.41049	-0.57907
N	-3.46129	3.20764	0.64747
C	-2.66962	2.23863	1.40695
C	-4.78014	2.64863	0.35277
H	-1.70149	2.65852	1.66740
H	-2.50469	1.30621	0.85454
H	-3.20274	2.01115	2.32856
H	-5.45205	3.41441	-0.02385
H	-5.20077	2.26013	1.27827
H	-4.73207	1.82428	-0.37247

5c

S	-2.16553	2.26622	-1.55838
C	-2.73293	3.62031	-0.40381
N	-1.40796	4.06570	0.05339
N	-0.33605	3.73443	-0.69024
N	-0.51626	2.81672	-1.54500
C	-1.25622	5.23503	0.90432
H	-2.05413	5.22166	1.64325
H	-1.30019	6.16876	0.34159
H	-0.29447	5.16714	1.40631
N	-3.51102	4.60178	-1.13705
N	-3.50278	3.14814	0.72724
C	-2.76823	2.25589	1.62381
C	-4.74859	2.49883	0.30825
H	-1.88296	2.74666	2.01690
H	-2.45950	1.32828	1.12750
H	-3.42812	2.00859	2.45397
H	-5.29395	3.13541	-0.38063
H	-5.36048	2.31423	1.18999
H	-4.55295	1.53626	-0.18416
C	-2.78798	5.23070	-2.23751
C	-4.22568	5.61767	-0.35680
H	-4.67012	5.17576	0.52780
H	-5.01563	6.03033	-0.98511
H	-3.58030	6.44676	-0.04469
H	-2.35052	4.47741	-2.88641
H	-1.99094	5.90233	-1.89144
H	-3.49626	5.81761	-2.82063

6a

N	0.56911	-0.48094	1.28056
C	1.85410	-1.03887	1.30167
C	2.31062	-1.50627	0.13112
S	1.18317	-1.22368	-1.19737
C	0.16345	-0.06948	-0.06842
N	-1.26298	-0.21608	-0.19597
C	-1.75065	-1.56498	0.09504
C	-1.78650	0.23570	-1.48208
C	0.61664	1.36167	-0.35619
H	0.41897	0.26285	1.95018
H	2.35897	-1.12973	2.25160
H	3.22477	-2.05390	-0.02139
H	-1.34934	-1.90056	1.04526
H	-1.46250	-2.28449	-0.68097
H	-2.83741	-1.52703	0.15750
H	-1.35391	-0.31722	-2.32875
H	-1.60861	1.29739	-1.62645
H	-2.86271	0.07306	-1.48943
H	0.51065	1.62174	-1.40652
H	1.67170	1.44234	-0.09763
H	0.03566	2.05932	0.25236

6a’

N	-0.53672	0.97641	-0.43896
C	0.29742	1.96072	0.09391
C	1.26743	1.54752	0.92512
S	1.30090	-0.19759	1.13308
C	-0.03204	-0.36856	-0.33528
N	0.56146	-0.91154	-1.52442
N	-1.03423	-1.34207	-0.03448
C	1.80302	-0.34404	-2.01771
C	0.38421	-2.30445	-1.46174
C	-1.71773	-1.28068	1.24310
C	-0.58114	-2.56498	-0.55857
C	-1.32844	1.27549	-1.62474
H	0.07790	2.98860	-0.15419
H	1.94780	2.19058	1.45722
H	2.00216	-0.75162	-3.00762
H	2.64467	-0.55685	-1.35456
H	1.69275	0.73541	-2.10289
H	0.89220	-2.96803	-2.13941
H	-1.06209	-1.55107	2.07489
H	-2.57204	-1.95500	1.21310
H	-2.07413	-0.26487	1.39772
H	-1.06674	-3.49385	-0.31647
H	-0.72254	1.37125	-2.53070
H	-1.86760	2.20665	-1.45620
H	-2.04827	0.47399	-1.77267

6b

N	-1.37091	-0.05411	-0.17220
N	-2.25860	-1.07475	0.16813
C	-1.90268	-1.66043	1.26093
S	-0.42032	-1.20491	2.05092
C	-0.01563	-0.16309	0.31580
N	0.93309	-0.84292	-0.47975
C	0.74213	-2.23958	-0.82377
C	2.19217	-0.31167	-0.17034
N	0.61778	1.06328	0.59201
C	0.04247	1.98257	1.55300
C	1.99687	0.85407	0.48303
H	-1.38847	0.08522	-1.17435
H	-2.53630	-2.43643	1.66502
H	-0.26100	-2.37517	-1.22260
H	0.86781	-2.88822	0.04583
H	1.46293	-2.51045	-1.59241
H	3.10019	-0.76131	-0.53058
H	0.55767	2.93753	1.47270
H	-1.00905	2.11997	1.31668
H	0.12733	1.59854	2.57287
H	2.70533	1.60239	0.79022

6b’

N	0.95732	-0.12749	-0.08582
N	1.79258	-1.11920	-0.58346
C	1.45275	-1.47607	-1.77697
S	0.03569	-0.78223	-2.51138
C	-0.37521	-0.06513	-0.64126
N	-1.40135	-0.82111	-0.02068
C	-1.31753	-2.26849	0.03756
C	-2.61039	-0.15485	-0.25742
N	-0.92524	1.23433	-0.68788
C	-0.26433	2.27258	-1.45334
C	-2.31971	1.09841	-0.66584
C	1.02886	-0.01235	1.36770
H	2.05917	-2.20093	-2.29959
H	-0.33683	-2.55485	0.41095
H	-1.46576	-2.72186	-0.94498
H	-2.07496	-2.63348	0.72878
H	-3.55894	-0.60496	-0.02368
H	0.78737	2.29112	-1.18034
H	-0.71799	3.23025	-1.20566
H	-0.34441	2.09082	-2.52848
H	-2.96908	1.93705	-0.84307
H	0.45362	0.86047	1.66845
H	2.07228	0.12543	1.63789
H	0.63629	-0.89219	1.88452

6c

N	-0.05721	1.76186	0.72236
N	0.83999	2.68799	0.30291
N	1.96921	2.24549	-0.05379
S	2.06556	0.51155	0.03227
C	0.18515	0.37354	0.39029
N	-0.59514	-0.13448	-0.70652
N	-0.16109	-0.53057	1.45164
C	-0.31213	0.34775	-2.04740
C	-0.78662	-1.50752	-0.46073
C	0.63413	-0.52851	2.66576
C	-0.52327	-1.74577	0.83711
C	-1.43387	2.22067	0.84597
H	-1.12777	0.04938	-2.70371
H	0.63241	-0.03545	-2.44023
H	-0.26539	1.43592	-2.03092
H	-1.19424	-2.16079	-1.21215
H	1.64113	-0.92387	2.50585
H	0.12129	-1.12984	3.41418
H	0.71633	0.49225	3.03246
H	-0.66860	-2.64057	1.41626
H	-1.93790	2.27200	-0.12028
H	-1.41708	3.20456	1.30530
H	-1.96463	1.52069	1.48633

6d

O	-0.84136	1.09654	-0.08978
C	-0.06705	2.00856	0.58982
C	0.87411	1.56129	1.42454
S	0.97044	-0.18317	1.51947
C	-0.55514	-0.25700	0.17548
N	-0.18923	-0.99367	-0.97094
N	-1.65330	-0.97807	0.69034
C	1.03770	-0.67722	-1.67348
C	-0.62025	-2.31189	-0.76190
C	-2.19318	-0.64263	1.99229
C	-1.51242	-2.30238	0.25047
H	-0.32001	3.03020	0.36005
H	1.52418	2.20340	1.99561
H	1.03506	-1.19776	-2.62902
H	1.92221	-0.96019	-1.09834
H	1.06618	0.39360	-1.86327
H	-0.33078	-3.10972	-1.42212
H	-1.51391	-0.92281	2.80056
H	-3.14601	-1.15350	2.11540
H	-2.36852	0.43028	2.03272
H	-2.13833	-3.09048	0.62909

6e

S	-1.33481	1.48539	0.00000
C	-0.08603	2.75552	0.00000
C	1.23305	2.47700	0.00000
S	1.89676	0.86541	0.00000
C	-0.33967	-0.03594	0.00000
N	-0.41338	-0.88409	-1.10349
N	-0.41338	-0.88409	1.10349
C	-0.21708	-0.40206	-2.46026
C	-0.17173	-2.18775	-0.68019
C	-0.21708	-0.40206	2.46026
C	-0.17173	-2.18775	0.68019
H	-0.50036	3.76319	0.00000
H	1.94326	3.30863	0.00000
H	-0.44340	-1.21254	-3.16300
H	0.82320	-0.05897	-2.58500
H	-0.89535	0.44070	-2.64385
H	-0.08205	-3.00760	-1.38245
H	0.82320	-0.05897	2.58500
H	-0.44340	-1.21254	3.16300
H	-0.89535	0.44070	2.64385
H	-0.08205	-3.00760	1.38245

7a

N	-1.15228	0.05398	0.26328
C	0.00000	0.00000	0.96553
C	-1.41021	-0.37802	-1.11206
C	-2.36949	0.25122	1.03939
N	1.15228	-0.05398	0.26328
C	2.36949	-0.25122	1.03939
C	1.41021	0.37802	-1.11206
H	-0.59959	-1.00541	-1.46778
H	-1.55493	0.45456	-1.80372
H	-2.32263	-0.97609	-1.11464
H	-2.96005	1.06995	0.62174
H	-2.08448	0.49311	2.05697
H	-2.98561	-0.65280	1.03405
H	2.96005	-1.06995	0.62174
H	2.08448	-0.49311	2.05697
H	2.98561	0.65280	1.03405
H	0.59959	1.00541	-1.46778
H	1.55493	-0.45456	-1.80372
H	2.32263	0.97609	-1.11464

7b

N	0.35125	-0.28207	0.02303
C	-0.71498	-1.02782	0.03836
C	-2.01216	-0.23895	-0.03391
F	-3.06052	-1.06410	-0.01182
C	1.65459	-0.95756	0.08983
F	-2.12357	0.49104	-1.17040
F	-2.17243	0.61127	1.00936
C	0.50115	1.18663	-0.05207
H	1.47433	-2.02382	0.14470
H	2.19437	-0.61326	0.97164
H	2.23446	-0.71119	-0.79928
H	1.03958	1.52266	0.83257
H	1.09060	1.42673	-0.93542
H	-0.45666	1.68044	-0.10658

7c

F	1.03076	0.00000	-0.26415
C	0.00000	0.00000	0.52836
F	-1.03076	0.00000	-0.26415

7d

C	0.00000	0.00000	0.98004
N	0.00000	-1.05978	0.11471
N	0.00000	1.05978	0.11471
C	0.00000	-2.43955	0.56740
C	0.00000	-0.68087	-1.21486
C	0.00000	2.43955	0.56740
C	0.00000	0.68087	-1.21486
H	0.88805	-2.95929	0.20970
H	-0.88805	-2.95929	0.20970
H	0.00000	-2.42361	1.65211
H	0.00000	-1.38415	-2.02879
H	-0.88805	2.95929	0.20970
H	0.88805	2.95929	0.20970
H	0.00000	2.42361	1.65211
H	0.00000	1.38415	-2.02879

7e

C	-0.13191	-1.13692	-0.04163
N	0.87581	-0.28450	-0.41185
C	2.26489	-0.49976	-0.20692
C	0.37523	0.93988	-1.04327
N	-1.33093	-0.56996	-0.38792
C	-2.61357	-1.14406	-0.18047
C	-1.17195	0.73869	-1.02861
H	2.41911	-1.49373	0.28998
H	2.69475	0.30959	0.44453
H	2.81029	-0.48926	-1.19002
H	0.67436	1.84560	-0.45538
H	0.76425	1.03863	-2.08932
H	-3.23759	-0.47861	0.47667
H	-2.49940	-2.14577	0.31169
H	-3.14743	-1.27289	-1.16153
H	-1.59254	0.73044	-2.06705
H	-1.68233	1.53986	-0.43451

8a

N	1.26911	-0.26133	0.50685
C	1.49057	-1.61218	0.21079
C	0.65073	-2.17090	-0.67428
S	-0.55844	-1.02259	-1.23739
C	-0.03102	0.24780	0.05837
N	-0.89832	0.44574	1.20077
C	-1.61837	-0.71179	1.70271
C	-1.68085	1.65396	0.98056
N	-0.03795	1.54017	-0.59859
C	1.17528	1.92932	-1.28817
C	-0.63984	2.51956	0.29330
H	1.42907	-0.00438	1.47196
H	2.33529	-2.10205	0.67147
H	0.70957	-3.17368	-1.06099
H	-0.92992	-1.54757	1.81776
H	-2.43063	-1.02298	1.03743
H	-2.03291	-0.46775	2.68070
H	-2.54249	1.48567	0.32024
H	-2.02916	2.05756	1.93066
H	0.96888	2.82541	-1.87326
H	1.47268	1.13322	-1.96825
H	2.00943	2.13191	-0.60638
H	0.06809	2.92568	1.03040
H	-1.06700	3.34203	-0.27962

8b

O	1.33982	-0.01858	0.83810
C	1.64535	-1.31031	0.52988
C	0.87610	-1.95086	-0.35711
S	-0.37859	-0.90065	-0.99353
C	0.11513	0.46291	0.21244
N	-0.81115	0.78927	1.24507
C	-1.66180	-0.25069	1.78478
C	-1.40546	2.08534	0.96133
N	0.28368	1.68249	-0.51716
C	1.56020	1.87484	-1.17824
C	-0.22761	2.77837	0.29710
H	2.50457	-1.69755	1.05385
H	1.00321	-2.96878	-0.68170
H	-1.06488	-1.13741	1.99021
H	-2.47726	-0.52904	1.10800
H	-2.08623	0.10054	2.72507
H	-2.25837	2.02133	0.27156
H	-1.72734	2.56518	1.88505
H	1.48225	2.73385	-1.84361
H	1.78612	0.99494	-1.77879
H	2.38105	2.04086	-0.47289
H	0.49402	3.12035	1.05052
H	-0.51566	3.61649	-0.33586

8c

S	0.95353	-0.13624	1.38265
C	1.72828	-1.52599	0.66205
C	1.04330	-2.22426	-0.25933
S	-0.53333	-1.65361	-0.75086
C	-0.48420	-0.00268	0.17074
N	-1.63392	0.34279	0.96162
C	-2.38170	-0.71389	1.61018
C	-2.38457	1.38432	0.27408
N	-0.41961	1.03739	-0.81149
C	0.86215	1.32604	-1.41985
C	-1.24316	2.15243	-0.36910
H	2.71766	-1.79721	0.99991
H	1.42376	-3.11325	-0.74124
H	-1.69540	-1.36027	2.15334
H	-2.95158	-1.32718	0.90220
H	-3.07087	-0.26170	2.32301
H	-3.06365	0.98750	-0.49295
H	-2.95520	1.96965	0.99409
H	0.70097	1.97275	-2.28174
H	1.30877	0.39524	-1.76721
H	1.55883	1.81415	-0.72956
H	-0.74067	2.79264	0.36905
H	-1.54761	2.75573	-1.22334

8d

N	-0.44112	-0.48179	1.11145
N	-0.10619	-1.82094	1.25285
C	0.26241	-2.33154	0.12849
S	0.19299	-1.29840	-1.27930
C	-0.73767	-0.00928	-0.25088
N	-2.15550	0.11948	-0.45411
C	-2.91288	-1.08665	-0.73722
C	-2.39489	1.27322	-1.30933
N	-0.21795	1.29184	-0.60922
C	0.93999	1.77103	0.11950
C	-1.33026	2.21612	-0.77609
H	-1.20243	-0.25804	1.73976
H	0.61003	-3.35164	0.07827
H	-2.66049	-1.85416	-0.00699
H	-2.71828	-1.48102	-1.74025
H	-3.97466	-0.86114	-0.64430
H	-2.22519	1.05372	-2.37223
H	-3.41158	1.64010	-1.17541
H	1.31384	2.66490	-0.37942
H	1.71961	1.01243	0.10003
H	0.71918	2.01123	1.16552
H	-1.65890	2.66670	0.17104
H	-1.06323	3.00905	-1.47388

	**8e**<br>N -1.27734 0.26255 0.95021<br>N -1.94131 -0.92510 0.72095<br>C -1.11876 -1.90225 0.53567<br>S 0.58304 -1.50810 0.55203<br>C 0.12047 0.32855 0.49325<br>N 0.26092 0.98830 -0.77488<br>C -0.07558 0.24879 -1.97640<br>C 1.51100 1.73130 -0.77436<br>N 1.02486 1.05121 1.35415<br>C 0.68821 1.17165 2.75704<br>C 1.52470 2.22836 0.66181<br>C -2.10575 1.43040 0.67678<br>H -1.48203 -2.90964 0.40708<br>H -1.04985 -0.22152 -1.85274<br>H 0.65867 -0.52798 -2.21594<br>H -0.13589 0.94858 -2.80986<br>H 2.38269 1.09215 -0.97348<br>H 1.47837 2.53215 -1.51233<br>H 1.56020 1.55289 3.28855<br>H 0.44165 0.19013 3.15553<br>H -0.15832 1.84307 2.94503<br>H 0.86986 3.10415 0.77180<br>H 2.52155 2.48586 1.01936<br>H -3.02017 1.32850 1.25617<br>H -2.35384 1.54002 -0.38125<br>H -1.57251 2.32094 1.00234	
